# Fluorescent Neutron Track Detectors for Boron-10 Microdistribution Measurement in BNCT: A Feasibility Study

**DOI:** 10.3390/ma18030621

**Published:** 2025-01-29

**Authors:** Laura Galuzzi, Gabriele Parisi, Valeria Pascali, Martin Niklas, Davide Bortot, Nicoletta Protti, Saverio Altieri

**Affiliations:** 1 Department of Energy, Politecnico di Milano, 20156 Milan, Italy; lauragaluzzi04@gmail.com (L.G.); davide.bortot@polimi.it (D.B.); 2 Department of Physics, University of Pavia, 27100 Pavia, Italy; valeria.pascali01@universitadipavia.it (V.P.); nicoletta.protti@unipv.it (N.P.); saverio.altieri@cnao.it (S.A.); 3 INFN—Istituto Nazionale di Fisica Nucleare, Sezione di Pavia, 27100 Pavia, Italy; 4 Division of Radiology and Division of Medical Physics in Radiation Oncology, DKFZ—Deutsches Krebsforschungszentrum, 69120 Heidelberg, Germany; m.niklas@dkfz-heidelberg.de

**Keywords:** Fluorescent Nuclear Track Detector, BNCT, boron microdistribution, particle track

## Abstract

Boron Neutron-Capture Therapy (BNCT) is a form of radiation therapy that relies on the highly localized and enhanced biological effects of the ^10^B neutron capture (BNC) reaction products to selectively kill cancer cells. The efficacy of BNCT is, therefore, strongly dependent on the ^10^B spatial microdistribution at a subcellular level. Fluorescent Nuclear Track Detectors (FNTDs) could be a promising technology for measuring ^10^B microdistribution. They allow the measurement of the tracks of charged particles, and their biocompatibility allows cell samples to be deposited and grown on their surfaces. If a layer of borated cells is deposited and irradiated by a neutron field, the energy deposited by the BNC products and their trajectories can be measured by analyzing the corresponding tracks. This allows the reconstruction of the position where the measured particles were generated, hence the microdistribution of ^10^B. With respect to other techniques developed to measure ^10^B microdistribution, FNTDs would be a non-destructive, biocompatible, relatively easy-to-use, and accessible method, allowing the simultaneous measurement of the ^10^B microdistribution, the LET of particles, and the evolution of the related biological response on the very same cell sample. An FNTD was tested in three irradiation conditions to study the feasibility of FNTDs for BNCT applications. The FNTD allowed the successful measurement of the correct alpha particle range and mean penetration depth expected for all the radiation fields employed. This work proved the feasibility of FNTD in reconstructing the tracks of the alpha particles produced in typical BNCT conditions, thus the ^10^B microdistribution. Further experiments are planned at the University of Pavia’s LENA (Applied Nuclear Energy Laboratory) to test the final set-up coupling the FNTD with borated cell samples.

## 1. Introduction

The interest in Boron Neutron-Capture Therapy (BNCT) has been recently increasing thanks to the latest advancements in accelerator technology, which allow the generation of the required neutron field from accelerator facilities that could be hosted in hospitals or clinical centers. BNCT is a binary form of particle therapy used in the treatment of tumor pathologies. It consists of two distinct phases: (i) the intravenous administration of a compound enriched in ^10^B, which selectively accumulates in tumor cells, followed by (ii) the irradiation with a thermal or epithermal neutron beam, depending on the depth of the tumor target [1]. Apoptosis of the tumor cells is triggered by significant DNA damage resulting from ionizations caused by the charged products of the ^10^B neutron-capture reactions, specifically an α particle and a ^7^Li nucleus. These products have ranges of approximately 9 µm and 4 µm, respectively, making them capable of depositing their energy primarily within the cell. The selectivity and effectiveness of BNCT thus depend closely on the biodistribution of ^10^B within the intracellular environment. Specifically, the likelihood of inducing DNA damage and subsequently killing the tumor cell increases with the greater internalization of ^10^B into the nucleus or its adjacent regions.

Currently, the two main ^10^B carriers employed for BNCT treatments are disodium mercaptoundecahydrododecaborate (BSH, Na_2_B_12_H_12_SH) and L-p-Boronophenylalanine (BPA, C_9_H_12_BNO_4_). The former accumulates in the intercellular space, while BPA is able to penetrate the cell membrane and potentially reach the nucleus. However, research on improved and optimized boron carrier compounds to improve BNCT clinical outcomes is very active. A comprehensive review of the state of the art of boron agents for BNCT can be found in [2]. Many different boron carriers have been studied by following different design strategies, including cell membrane targeting, nuclear targeting, and tumor affinity. It is evident from the described scenario that the biodistribution of ^10^B depends on the type of carrier administered to the patient and how this, in turn, is internalized by the individual cell. It is, therefore, crucial to study the ^10^B microdistribution at a subcellular level, both inter- and intracellularly, as it has a significant impact on the efficacy of the treatment.

Several studies are currently underway to develop techniques capable of measuring the microdistribution of ^10^B within cells using different methods [1,3]: secondary ion mass spectrometry (SIMS) [4,5,6,7,8,9] and Nano-SIMS [10,11], high-resolution alpha autoradiography [12,13,14,15], neutron autoradiography [16,17,18,19,20,21,22,23,24], electron energy loss spectroscopy (EELS) [25,26,27,28,29], laser secondary neutral mass spectrometry (SNMS) [6,30,31], laser-induced breakdown spectroscopy (LIBS) [32,33,34,35], stimulated Raman scattering (SRS) [36], nuclear magnetic resonance or magnetic resonance imaging (MRI) on protons [37,38,39] or on ^10^B [40] and immunohistochemistry [41]. Nano-SIMS, EELS, and SNMS are the techniques allowing for the best spatial resolution, as low as a few hundred nm and even lower in the case of EELS. However, they all require very complex set-up, procedures and specific facilities to be carried out. SIMS, besides its limits on sensitivity and quantification, necessitates measurement and analysis under vacuum conditions using highly focused low-energy ion beams and mass spectrometers [3]. SNMS and EELS are both time-, labor- and cost-intensive and require complex procedures for the preparation and analysis of cell samples [3]. They require vacuum conditions, cryo-fixation and cryo-sectioning of very thin frozen samples and cryo-analysis. Techniques such as LIBS and MRI have more accessible requirements in terms of sample preparation and analysis. However, MRI has a much lower spatial resolution and sensitivity, while LIBS requires a calibration curve relying on standard samples, which are difficult to produce from biological materials. While still under development, neutron autoradiography was proven a promising technique that is able to achieve subcellular spatial resolution for assessing how ^10^B is internalized and whether it reaches recognizable cell structures. However, to obtain such a resolution, the neutron autoradiography has to be overlapped with the histological image of the biological sample [22,23]. This is a critical step of the analysis procedure as it affects the measurement accuracy.

This work aims to study the feasibility of an experimental method relying on a Fluorescent Nuclear Track Detector (FNTD) [42]. This device has several key advantages, including excellent energy and spatial resolution, biocompatible surface, and reusability. FNTDs have already been applied in hadron therapy, with the FNTD being the central component of the biosensor Cell-Fit-HD^4*D*^ [43,44]. This study represents a significant innovation in the field of BNCT, as it is the first time that FNTD detectors have been explored as potential tools for measuring the microdistribution of ^10^B. To this end, an FNTD readout from a confocal laser-scanning microscope was tested in three different conditions to prove its capabilities of accurately measuring the particle tracks of boron neutron-capture (BNC) products. Hence, the device was first irradiated by the alpha particles emitted by an Am-241 source (with energy of about 5.5 MeV). The same radiation field moderated by a 23 μm layer of Mylar was then used to obtain alpha particles with a lower energy of about 2.5 MeV, thus being closer to BNC products. Finally, the FNTD was tested at the University of Pavia’s LENA (Applied Nuclear Energy Laboratory) under a thermal neutron beam. A standard reference material (SRM) consisting of boron implanted on a silicon wafer was placed on the detector surface to reproduce a typical BNCT radiation field.

## 2. Materials and Methods

The characterization of FNTD detectors was carried out in radiation fields relevant to BNCT by combining the results of different experiments with reference simulations. The particle transport was simulated by means of SRIM-2013 software [45] to describe the particle path in the detector.

### 2.1. The Fluorescent Neutron Track Detector

Fluorescent Neutron Track Detectors (FNTDs) are a well-established technology based on a single crystal made of aluminum oxide doped with carbon and magnesium impurities: $Al_{2}O_{3}:C,Mg$. The crystal is grown following the Czochralski technique [42]. Carbon and magnesium doping is obtained, respectively, by dissolving carbon monoxide and by adding magnesium compounds (such as MgO or $MgAl_{2}O_{4}$) in the raw material.

The single and double vacancies found in $Al_{2}O_{3}:C,Mg$ crystals result in different fluorescent centers: *F*-centers characterized by an absorption band at 205 nm and by an emission band at 420 nm with a 2 ns lifetime, $F^{+}$-centers absorbing at 230 and 255 nm and emitting at 330 nm with 2 ns lifetime, and $F_{2}^{2+}$-centers absorbing at 435 nm and emitting at 520 nm with about 9 ns lifetime. $F_{2}^{2+}$-centers can easily absorb free-electrons generated during irradiations, creating $F_{2}^{+}$-centers by photochromic and radiochromic transformations. $F_{2}^{+}$-centers are characterized by excitation bands at 335 and 620 nm and emission band at 750 nm with a lifetime of 75 ns. The very short emission lifetime of these crystals allows for a fast laser-scanning readout. To reduce background luminescence, FNTDs are thermally annealed with a 17 h long specific heating profile up to 650 °C and successively treated with optical bleaching. Annealing and optical bleaching can be repeated to erase fluorescent tracks, thus allowing the detector to be reused. FNTDs can measure particles with LET ranging from 0.5 to 1800 keV μm^−1^, allowing the reaching of a fluence of $5\times 10^{7}$ cm^−2^ without saturation and to sustain high dose-rate up to $1\times 10^{8}$ Gy s^−1^ [44].

From the $Al_{2}O_{3}:C,Mg$ crystal, a plate-shaped cut with typical dimensions 8×4×0.5 $mm^{3}$ is usually obtained, as for the FNTD employed in this work.

### 2.2. Readout System

The FNTD readout was carried out by Confocal Laser-Scanning Microscopy (CLSM). CLSM allowed a non-destructive readout with diffraction-limited spatial resolution. A Zeiss LSM710 ConfoCor 3 inverted CLSM (Zeiss AG, Oberkochen, Germany) was employed for the experiments. Data acquisition was managed using the ZEN software (version 2009). The microscope consists of a 63× oil objective and an Avalanche Photo-Diodes (APD) in Geiger mode, a main beam splitter (BS488/561/633), and a single long-pass emission filter (LP655) placed in front of the APDs for signal separation, and a 633 nm helium-neon laser excitation source. The pinhole diameter was set to 1 Air Unity (AU) to achieve an optimal trade-off between resolution, contrast, and brightness. The FNTD was mounted in a glass-bottom dish. Immersion oil was applied between the dish and the objective lens.

### 2.3. Data Processing

The images acquired were processed using Fiji (https://imagej.net/software/fiji/ (accessed on 15 January 2023)) [46], an image processing software package based on ImageJ (https://imagej.net/software/imagej/ (accessed on 15 January 2023)). The 3D image processing routine created a maximum intensity projection along the detector depth, allowing for the separation of each track, which was then processed individually. For each track, the signal of each depth-slice was fitted with a 2D Gaussian. This made it possible to obtain the fluorescence amplitude, the fluorescence background offset, the coordinates of the track centroid ($x_{0}$, $y_{0}$), the track maximum and minimum width ($W_{y}$ and $W_{x}$, respectively), and the azimuthal angle of the track propagation (φ). The radial extension of the track is the effect of secondary electrons emitted by the interaction of the particle tracking with the detector material.

**Figure 1:**
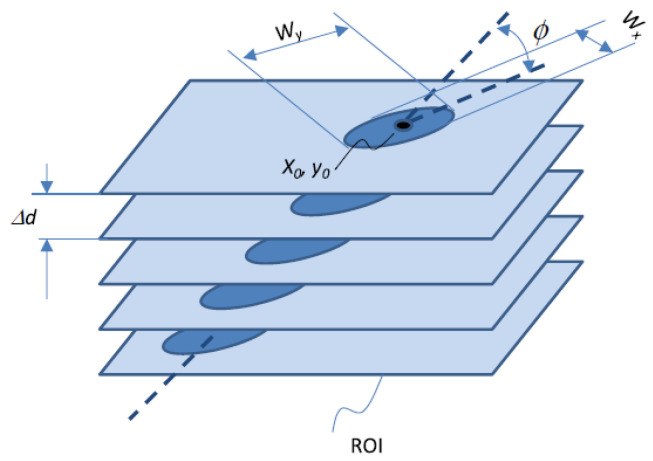
Diagram illustrating the data processing of a stack of images separated by a depth increment Δd for the region of interest (ROI) of an individual track. The typical value of Δd for the system employed is around 1 μm. The resulting quantities are represented.

Figure 1 schematically represents the quantities obtained through data analysis. From the fluorescence signals, the linear energy transfer (LET) of the ion traversing the FNTD can be assessed. In a BNCT context, if the particle type and emission energy are known, the position where the particle detected originated, thus the ^10^B position in the analyzed sample, can be estimated. This is achieved by combining the track direction measured, the energy released into the detector, the particle LET into the sample material, and the emission energy. However, only the track length and direction were considered in this work to study the feasibility of FNTD detectors for BNCT applications.

### 2.4. The Experiments

Three experiments were carried out to characterize the FNTD readout signal in BNCT-relevant radiation fields, thus to assess its feasibility for BNCT applications.

Two preliminary tests were carried out employing an Am-241 radioactive source. The three most probable decay channels of Am-241 emit α particles at 5.486, 5.442, and 5.388 MeV in the 84.8%, 13.1% and 1.6% of emissions, respectively. The source, disk-shaped with 4 mm diameter, had an activity of 2638 Bq to the irradiation day. The measurement was carried out by placing the detector at 1.16 cm from the source inside a vacuum chamber. A fluence of $4.466\times 10^{5}$ cm^−2^ was estimated.

Since the boron neutron capture produces α particles at 1.472 MeV in the 93.7% of reactions or at 1.776 MeV in the 6.3%, a second test was carried out to better reproduce such irradiation condition. With this purpose, a 23 μm layer of Mylar ($H_{8}C_{1}0O_{4}$) was interposed between the Am-241 source and the FNTD. The Mylar layer worked as a moderator, lowering the energy of the α particles emitted to about 2.5 MeV. In addition, a hole was made in the center of the Mylar layer to assess the capability of the FNTD to discriminate between the two resulting α energies (about 5.5 and 2.5 MeV).

Following the successful results of the two preliminary tests, the FNTD was eventually used to measure the radiation field produced by the boron neutron-capture reaction.

**Figure 2:**
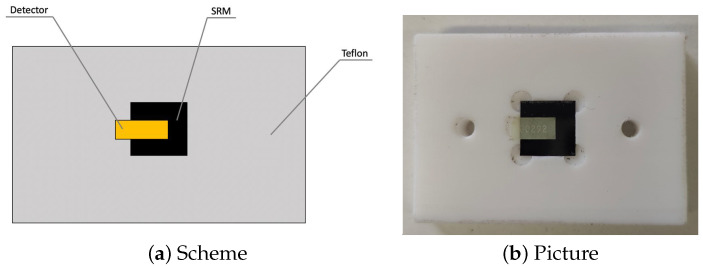
Schematic representation and picture of the experimental set-up used for the measurement of the SRM-2137 sample using the FNTD.

A sample of the standard reference material (SRM) SRM-2137 [47], certificated by the National Institute of Standards and Technology (NIST), was irradiated by a thermal neutron field in the thermal column of the TRIGA Mark II research reactor of University of Pavia’s LENA (Applied Nuclear Energy Laboratory). The SRM-2137 consists of a single-crystal silicon substrate with a $1.018\;\cdot \;10^{15}\pm 0.035\;\cdot \;10^{15}$ $\mathrm{atoms}/cm^{2}$ superficial concentration of ^10^B obtained by ion implantation. As shown in Figure 2, the FNTD was placed in contact with the SRM sample inside a Teflon structure, which held them in position. The low neutron activation of Teflon allowed for the minimization of the background radiation generated by the holder structure. A portion of FNTD was kept outside the SRM sample to observe and evaluate the background resulting from neutron irradiation. The irradiation was carried out with a reactor nominal power of 10 kW. The irradiation time was evaluated in order to allow the measurement of an α fluence of about 5×10^5^ cm^−2^.

## 3. Results

### 3.1. Preliminary Tests with Am-241

**Figure 3:**
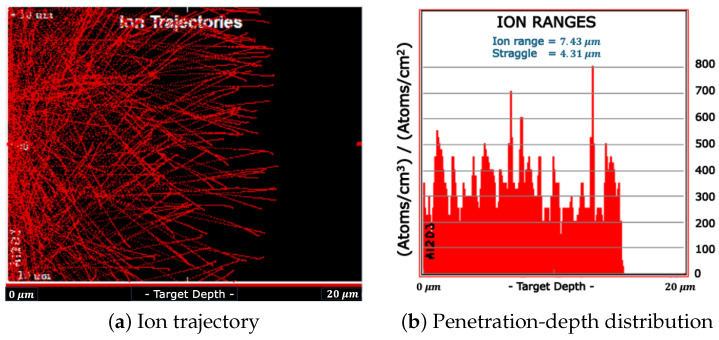
SRIM Simulation of the α particles isotropically emitted by an Am-241 source as they travel into the FNTD.

**Figure 4:**
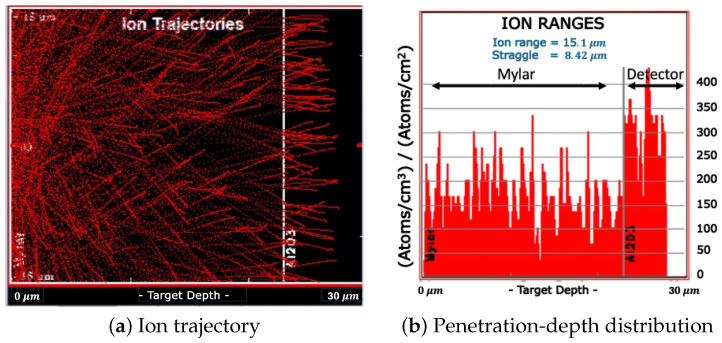
SRIM Simulation of the α particles isotropically emitted by an Am-241 source as they travel into a 23 μm Mylar layer and successively into the FNTD.

The results of SRIM simulations are shown in Figure 3 and Figure 4 for the measurement without and with the Mylar layer, respectively.

The simulations were carried out considering an isotropic surface source of α particles, reproducing the Am-241 emission. The thickness of the Am-241 source was not considered in the simulations. Figure 3b and Figure 4b represent the distribution of α particles as a function of their penetration depth inside the FNTD. In the case of isotropically emitted α without a Mylar layer, their penetration depth was evenly distributed between 0 and about 15 μm. 15 μm corresponds to the particle range in the FNTD material (i.e., the penetration depth of those particles emitted in the detector-axis direction). The mean penetration depth was 7.43 μm. When the 23 μm layer of Mylar is interposed between the Am-241 source and the detector, as clearly shown in Figure 4b, a significant number of particles was not able to reach the FNTD detector. According to the results of SRIM simulations, the α particles that were capable of reaching the detector had a mean energy of about 2.25 MeV and could penetrate up to 5 μm into the FNTD detector.

**Figure 5:**
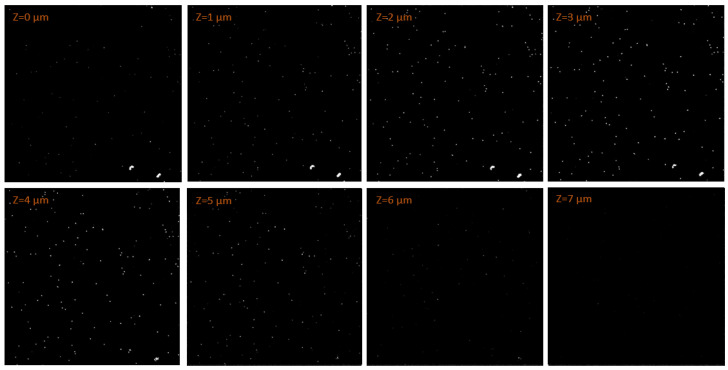
Fluorescent tracks images measured by the CLSM from a 135×135 $\mu m^{2}$ region of the FNTD at different depths. The FNTD was irradiated in a vacuum chamber with the α particles emitted by an Am-241 source.

The results of the experiments well agreed with what was expected from the simulations. The preliminary experiments also proved the capability of the FNTD detector read by the CLSM to measure the tracks of the α particle produced by Am-241, both with and without the 23 μm Mylar layer moderation. Figure 5 shows an example of the α tracks measured in a subregion of the FNTD at different depths in the case of no moderator.

Dirt on the FNTD surface, such as the two brightest features at the bottom-right of the FNTD region shown in Figure 5, was excluded from data analysis by properly setting a threshold system. After reaching a maximum brightness at about 4 μm, the majority of the tracks measured vanished at a depth of around 7 μm into the FNTD. This was in agreement with the mean penetration depth predicted by the simulations. The tracks had an oval shape as their direction was predominantly different from the detector axis due to the isotropic emission. The average track area resulted 0.329 μm^2^, corresponding to an equivalent diameter of approximately 0.5 μm. The result obtained well aligned with previous studies in literature [48]. The fluence resulting from the measurement was 6.1×10^5^ cm^−2^, which agreed within uncertainty to the nominal fluence expected of 4.466×10^5^ cm^−2^.

**Figure 6:**
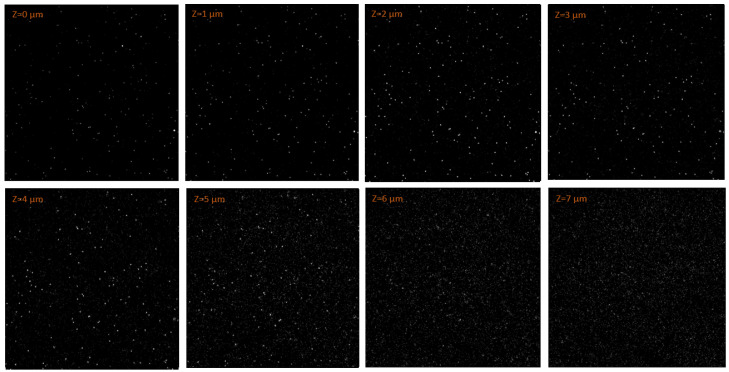
Fluorescent tracks images measured by the CLSM from a 135×135 $\mu m^{2}$ region of the FNTD at different depths. The FNTD was irradiated in a vacuum chamber with the α particles emitted by an Am-241 source and moderated by 23 μm thick Mylar layer.

**Figure 7:**
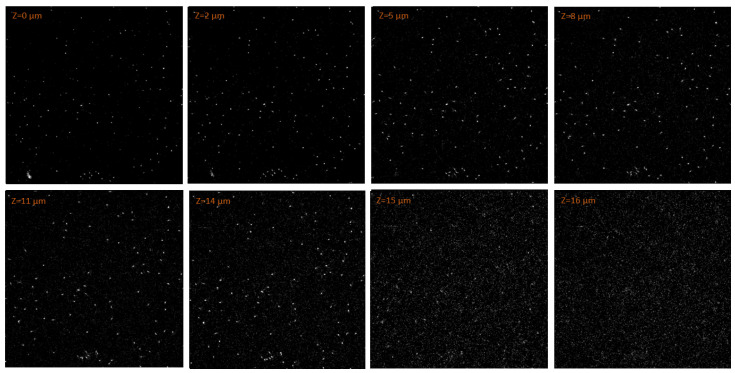
Fluorescent tracks images measured by the CLSM from a 135×135 $\mu m^{2}$ region of the FNTD at different depths. The FNTD was irradiated in a vacuum chamber with the α particles emitted by an Am-241 source and collimated by the perforated Mylar layer.

The measurement of the Am-241 source moderated by a 23 μm thick Mylar layer is reported in Figure 6 and Figure 7. They show, respectively, a subregion of the FNTD beneath the Mylar layer and a subregion beneath the central hole.

In both cases, the resulting track size was consistent with that measured in the case without a moderator. In the subregion beneath the Mylar, most of the tracks could penetrate to 4–5 μm, and almost none could reach 6 μm, as predicted by the simulations. In correspondence with the central hole, as expected, the tracks measured were all able to penetrate down to 14 μm into the FNTD. The number of α that were detected at 15 μm significantly dropped and almost zeroed at 16 μm. The Mylar layer, indeed, basically worked as a collimator in the central hole region. Considering the small diameter of the hole with respect to the Am-241 source, only those non-moderated α particles with a direction similar to the detector axis (perpendicular to the detector surface) were able to reach the detector in the hole region. As already discussed, the penetration depth of such α particles could be reasonably approximated with their range, 15 μm.

**Figure 8:**
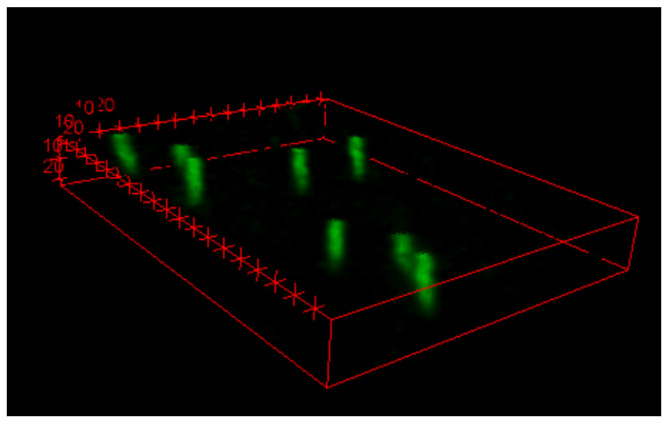
3D reconstruction of the α particles measured by the FNTD when emitted by the Am-241 source without any moderator. The unit of length on the three axes is the μm, while each tick mark corresponds to 10 μm.

The results obtained also allowed the reconstruction of the 3D particle track, as shown by Figure 8.

### 3.2. Measurement of a Reference Boron Neutron-Capture Therapy Radiation Field

Boron neutron capture has two reaction channels. The first occurs with the 93.7% of probability and produces a 1.472 MeV α particle, a 0.84 MeV lithium ion and a 478 keV γ-ray. The second reaction channel occurs with the 6.3% of probability and produces a 1.776 MeV α particle and a 1.015 MeV lithium ion. While the α particles are characterized by a range in the FNTD material of 2.9 μm (at 1.472 MeV) and 3.54 μm (at 1.776 MeV), the lithium ions have a range of just 1.52 μm (at 0.84 MeV) and 1.88 μm (at 1.015 MeV). Since the α and the lithium produced by the neutron reaction are emitted isotropically in opposite directions, the FNTD detects a comparable amount of the two particles. The readout and data analysis were significantly more complicated than in the experiment with the Am-241, especially within the first 2 μm of detector-sensitive volume due to the radiation field heterogeneity. However, since lithium has a higher atomic number than helium, its corresponding track spot should be of greater diameter and of higher intensities (because of the higher ionization density and stopping power). Hence, a meticulous analysis might allow the discrimination between the two particles, especially when their impinging angle is perpendicular to the detector surface, and their track shape is circular. When considering an isotropic emission of α and lithium with boron neutron-capture reaction energies and probabilities, the mean penetration depth resulting from SRIM simulations was 1.46 μm for α particles and 0.81 μm for lithium ions.

**Figure 9:**
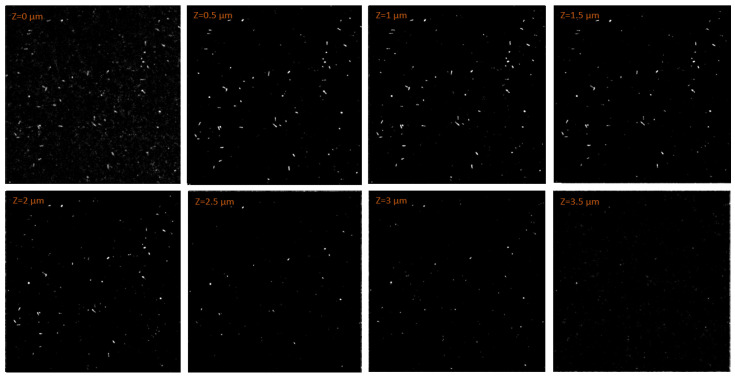
Fluorescent tracks images measured by the CLSM from a 67.5×67.5 $\mu m^{2}$ region of the FNTD at different depths. The FNTD measured the radiation field obtained by irradiating a sample of NIST SRM-2137 with a thermal neutron field.

The data processing was further complicated by the high background created by the neutron field and by the photons produced in the reactor. Nevertheless, thanks to the background data collected, allowing for an accurate setting of thresholds and for clean CLSM images, the tracks of boron neutron-capture reaction products were successfully discriminated from the background. Figure 9 shows the particle tracks measured by a 67.5 × 67.5 $\mu m^{2}$ subregion of the FNTD. The results of the experiment were in agreement with what was predicted by the simulations. The higher density of tracks was observed within the first μm of the FNTD, where the radiation field was composed of both reaction products. The number of tracks measured deeper than 2 μm significantly dropped, reasonably in line with the α mean penetration depth simulated. Eventually, only very few tracks were found at a depth of 3.5 μm, all characterized by a circular shape. These were the few 1.776 MeV α particles which entered the FNTD parallel to the detector axis and whose penetration depth thus corresponded to their range in the FNTD material.

## 4. Discussion

While a clear 3D reconstruction of the α tracks was obtained for the experiments with the Am-241 source, the higher background generated in the LENA’s reactor significantly complicated the 3D track reconstruction for the experiment with the SRM. Additional efforts should be put into the improvement of the radiation background to allow a clearer reconstruction of 3D tracks even in this experimental condition. However, this limitation did not hinder the successful visualization and measurement of particle tracks on 2D planes at different depths in the FNTD. This allowed the measurement of the track shape, size, length, and direction. Hence, despite the challenges posed by the high radiation background, the quantities required to estimate the emission point of the reaction products could still be measured by the FNTD.

A spatial resolution of a few μm was obtained for the particle track measured. This will allow, once the FNTD detector is coupled with borated cell samples, for the measurement of the ^10^B microdistribution with a subcellular spatial resolution. Table 1 summarizes the qualitative spatial resolution achieved by the different ^10^B microdistribution measurement techniques available in the literature. The resolution of FNTDs could be increased to approximately 80 nm if optical nanoscopy is used for the readout [49]. The final stage of FNTD systems for ^10^B microdistribution measurement will be their use with cell samples and the creation of the biosensor ${\mathrm{Cell}-\mathrm{Fit}-\mathrm{HD}}^{4D}$ by coupling the FNTD with time-lapse microscopy. Besides its promising spatial resolution, the main advantages of using FNTD detectors are their capability to simultaneously measure the ^10^B microdistribution, the particle track, and energy deposition and to follow the evolution of biological effects. This would be a unique feature of FNTD systems, which would allow the study, on the very same sample of cells, of the ^10^B microdistribution and the evolution of the biological effects induced by the BNC reactions, directly linking them with the LET of the particles causing them. In addition, unlike other techniques, FNTDs allow a non-destructive analysis and are relatively easy to use and easily accessible, as they rely on equipment that is usually available in radiation laboratories.

## 5. Conclusions

Fluorescent Neutron Track Detectors (FNTDs) can have a pivotal role in Boron Neutron-Capture Therapy (BNCT) as they allow the measurement of the ^10^B microdistribution in tumor cells. This work presented a feasibility study and the very first characterization of an FNTD for BNCT. The detector was read by Confocal Laser-Scanning Microscopy (CLSM). To reproduce a typical BNCT radiation field, the FNTD was employed in three experimental conditions: using an Am-241 source, using the same Am-241 source moderated by a 23 μm thick Mylar layer, and using the NIST Standard Reference Material (SRM) 2137 irradiated by a thermal neutron field. The third experiment was carried out at the University of Pavia’s LENA (Applied Nuclear Energy Laboratory), where the system made of the FNTD and the SRM-2137 sample was irradiated in the research reactor thermal column. The FNTD successfully measured the range and mean penetration depth of the α particles in all the experimental conditions tested. Hence, the results of this work proved the feasibility of FNTDs to reconstruct the tracks of the reaction products created in typical BNCT conditions.

By proving the track reconstruction capability of FNTDs for boron neutron-capture products, this work proved the feasibility of this type of detector to measure the ^10^B microdistribution in a material sample placed on the detector surface. Once the particle track is reconstructed, indeed, the particle emission point can be estimated by employing existing accredited software. The emission point coincides with the ^10^B location, and its microdistribution could finally be estimated.

To characterize the ^10^B microdistribution at the cell level, future experiments will be carried out, depositing a layer of ^10^B enriched cells on the FNTD. This hybrid detector, known as *Cell-Fit-HD*, can be coupled with time-laps microscopy to create the biosensor Cell-Fit-HD^4*D*^. Tracking specific proteins responsible for DNA repair, Cell-Fit-HD^4*D*^ will allow the study of the correlation between DNA damage dynamics and the stochastic energy deposition at a subcellular level. The development of such a technique will be pivotal in BNCT research, as it will allow the experimental study of the double stochastic nature of radiation interaction involved in BNCT: the ^10^B position with respect to the cell nucleus and the stochastic nature of energy deposition.

## Figures and Tables

**Table 1:** Summary of the qualitative spatial resolution of different techniques for the ^10^B microdistribution measurement. Most of the estimates are derived from the discussions in [1,3].

Technique	Qualitative Spatial Resolution	
FNTD	Subcellular	μm
EELS	Subcellular	tens of nm
SNMS	Subcellular	hundreds of nm
SIMS	Subcellular	a few μm
Nano-SIMS	Subcellular	hundreds of nm
Autoradiography	Subcellular	μm
LIBS	Cellular/Subcellular	10 μm
MRI	Organs	

## Data Availability

The original contributions presented in this study are included in the article. Further inquiries can be directed to the corresponding author.
